# Trends of obesity rates between primary total hip arthroplasty patients and the general population from 2013 to 2020

**DOI:** 10.1186/s42836-022-00140-9

**Published:** 2022-09-08

**Authors:** Nishanth Muthusamy, Thomas Christensen, Vivek Singh, Chelsea Sue Sicat, Joshua C. Rozell, Ran Schwarzkopf, Claudette M. Lajam

**Affiliations:** Department of Orthopedic Surgery, Division of Adult Reconstructive Surgery, NYU Langone Health, 301 East 17th Street, NY 10003 New York, USA

**Keywords:** Body mass index (BMI), Obesity, Total hip arthroplasty, Annual physicals

## Abstract

**Background:**

The prevalence of obesity in total hip arthroplasty (THA) patients has been studied in the past. However, there has not been direct comparison against obesity in the general population. This study compared yearly trends in BMI and obesity rates between patients who had undergone primary THA and those from the general patient population.

**Methods:**

We retrospectively reviewed all patients over the age of 18 who underwent primary, elective THA and those who had an annual routine physical exam between January 2013 and December 2020 at our academic tertiary medical center. Baseline demographics were controlled in our statistical models. Significance of yearly trends was determined through a linear regression analysis. Independent samples *t*-test and Chi-square test were used to compare means and proportions between the two groups, respectively.

**Results:**

A total of 11,250 primary THA patients and 1,039,918 annual physical exam patients were included. Average BMI for the THA group was significantly higher (*P* < 0.001) each year compared to the annual physicals group (APG). Higher obesity rates were observed in all obesity subgroups (all classes, and class I–III individually) for THA patients each year compared to the APG. Interestingly, while we found a significantly increasing trend in obesity for the general population (*P* < *0.001*), BMI and obesity rates remained stable in the THA population.

**Conclusion:**

While our general patient population showed significant increase in BMI and obesity over time, THA patients had higher, yet stable, BMI. Further investigation is required to determine the role of risk optimization in these findings.

**Level III Evidence:**

Retrospective Cohort Study.

## Background

Obesity, defined as a body mass index (BMI) ≥ 30 kg/m^2^, is endemic in the United States (U.S) as its prevalence rate increased from 30.5% in 1999 to 42.4% in 2018 [1, 2]. Moreover, obesity is a risk factor for severe hip osteoarthritis, possibly contributing to the rise in demand for total hip arthroplasty (THA) [3, 4]. One study reported that compared with a general patient population, patients with obesity had 3.42 times, 5.24 times, and 8.56 times higher risk of undergoing THA for obesity class I, II, and III, respectively [3]. The risk of complications such as infection, dislocations, and reoperations following THA is also higher in patients with obesity [4].

To understand the magnitude of its impact, researchers have evaluated trends in obesity rates for the total hip arthroplasty (THA) population at the national level using Nationwide Inpatient Sample (NIS) and the American College of Surgeons National Surgical Quality Improvement Program (ACS-NSQIP) [1, 5, 6]. Studies since 1993 have all found increases in prevalence of obesity in THA patients over time [1, 5, 6]. Pirruccio *et al*. [5] also reported BMI in THA patients from 2008–2016 to be significantly higher when compared to the overall U.S adult population. Furthermore, Singh *et al*. [7] analyzed trends from 1993–2005 at their institution and found similar results: BMI and obesity rates in THA patients increased significantly over this period.

Despite these results, the advent of value-based payments in 2013 with emphasis on medical optimization and weight assessment may have influenced both patient selection and weight optimization for THA patients. Therefore, it is important to continue to track how obesity rates in the THA population compare to those of the general patient population not receiving THA. Evaluating the current trends over time enables surgeons to better understand the relationship between weight and arthritis progression, and whether optimization of THA patients requires additional focus on weight management. This study analyzed the trends in BMI and obesity rates in patients who had undergone primary THA at a large urban center against the general population of patients at the same institution. Our aim was to provide THA surgeons with the most recent information on obesity trends in order for them to internally assess the impact of current preoperative optimization strategies on patient BMI. Furthermore, our data may assist with shaping institutional guidelines for patients with obesity who may be candidates for THA. Subsequently, our results will provide better guidance for surgical teams to minimize risk following THA.

## Materials and methods

### Study design

We retrospectively analyzed patient data from a single, academic, orthopedic specialty hospital. The study population from our institution was stratified into two cohorts: (1) those who underwent elective primary THA between January 2013 and December 2020 and (2) all patients who had an annual routine physical exam and had not undergone a primary THA within the same period. Annual physical exams were identified using the Current Procedural Terminology (CPT) codes 99385, 99386, 99387, 99395, 99396, and 99397. Patients under 18 and those who underwent non-elective surgery such as revision THA or primary THA for hip fracture were excluded from this analysis. Approval from our Institutional Review Board (IRB) was obtained prior to conducting this study.

### Data collection

We collected baseline demographic variables which included age, sex, and BMI. All data were extracted using our institution's electronic medical records database (Epic Caboodle, version 15; Verona, WI, USA) and were de-identified and encrypted with Microsoft Excel software. The primary outcomes were average BMI and yearly trends between patients undergoing primary THA and those who had routine physical exams. We then separated the study population into five categories based on the CDC classification of obesity [8]: underweight (BMI: < 18.5 kg/m^2^), all obese (BMI: ≥ 30 kg/m^2^), Class I obesity (BMI: 30–34.9 kg/m^2^), Class II obesity (BMI: 35–39.9 kg/m^2^), and Class III obesity (BMI: ≥ 40 kg/m^2^). The secondary outcome was the yearly trends in obesity rate between the two cohorts in each sub-group. Finally, we grouped study patients into matched age subgroups and compared average BMI between the two populations within each range. There was no funding source for the study.

### Statistical analysis

Statistical analyses were performed using SPSS v25 (IBM Corporation, Armonk, New York, USA). Baseline characteristics such as age and gender were first compared using multilinear regression to ensure that these factors were statistically equivalent between the two cohorts. Pearson’s Chi-square (χ^2^) tests were utilized to detect statistical differences in categorical variables while independent sample two-sided *t*-tests were used for continuous variables. Furthermore, additional descriptive statistics were presented as means ± standard deviation for BMI and total counts (%) for obesity rates. A linear regression was used to calculate the difference in means for BMI and the unstandardized beta values. Linear regression with Pearson’s correlation coefficient (r) was used to determine the significance of the yearly trends for both groups. A significant slope (Pearson’s r) indicated an increasing or decreasing trend, while a lack of significance indicated a stable trend. A *P*-value of less than 0.05 was set as statistically significant.

## Results

This study included 11,250 primary THA patients and 1,039,918 annual physical exam (APG) patients. Comparison of baseline characteristics between the two groups found differences in age and gender; patients in the primary THA group were older (63.08 years ± 11.56 years *vs*. 44.17 years ± 14.99 years, *P* < 0.001) and had higher percentage of males (43.0% *vs*. 33.5%, *P* < 0.001) than the APG group (Table 1).

**Table 1:** Patient Population Comparison (2013–2020)

	**THA (*n* = 11,250)**	**Annual Physicals (*n* = 1,039,918)**	***P*-value**
**Age (years)**	63.08 ± 11.56	44.17 ± 14.99	< 0.001
**Gender**			< 0.001
Female	6,416 (57.0%)	691,257 (66.5%)	
Male	4,834 (43.0%)	348,661 (33.5%)	

After adjusting for demographics, there were significant differences in average BMI between groups each year. Mean BMI for the THA group was significantly greater (*P* < 0.001) each year compared to the APG (Table 2).

**Table 2:** Trends of BMI 2013–2020 (BMI; kg/m^2^)

	**THA**	**Annual Physicals**	**Unstandardized Beta (95% CI)**	***P*-value**
**2013**	29.3 ± 6.00 (*n* = 832)	25.39 ± 5.33 (*n* = 23,837)	-1.65 (-2.03 to -1.27)	< 0.001
**2014**	29.07 ± 5.89 (*n* = 1,109)	25.70 ± 5.40 (*n* = 39,341)	-1.44 (-1.76 to -1.12)	< 0.001
**2015**	28.97 ± 6.06 (*n* = 1,379)	26.30 ± 5.58 (*n* = 66,708)	-1.04 (-1.33 to -0.74)	< 0.001
**2016**	28.91 ± 6.25 (*n* = 1,584)	26.59 ± 5.75 (*n* = 99,086)	-0.82 (-1.10 to -0.53)	< 0.001
**2017**	28.77 ± 5.85 (*n* = 1,709)	26.90 ± 5.88 (*n* = 147,724)	-0.43 (-0.71 to -0.15)	< 0.001
**2018**	28.93 ± 5.93 (*n* = 1,777)	27.36 ± 6.04 (*n* = 214,520)	-0.32 (-0.60 to -0.04)	< 0.001
**2019**	29.34 ± 6.07 (*n* = 1,547)	27.47 ± 6.06 (*n* = 236,238)	-0.72 (-1.02 to -0.42)	< 0.001
**2020**	29.38 ± 6.03 (*n* = 1,313)	27.64 ± 6.13 (*n* = 212,464)	-0.72 (-1.06 to -0.39)	< 0.001

**Fig. 1:**
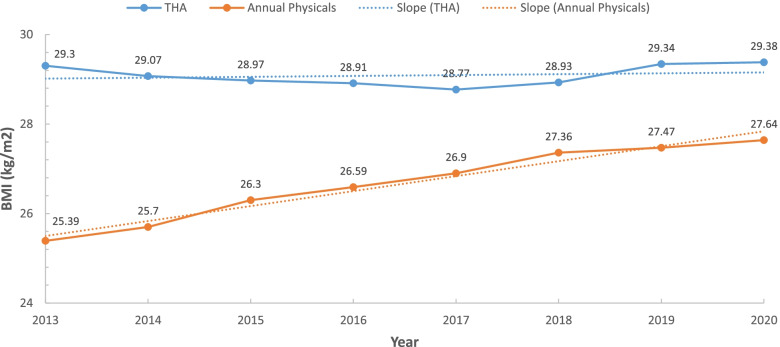
BMI Trends (THA *vs*. Annual Physicals)

Furthermore, BMI trends analysis showed a significant positive slope (*P* < 0.001) for the APG, indicating an increasing BMI trend for our general population (Fig. 1, Table 3). Conversely, the slope of the THA group was not significant and was essentially flat. This indicated that BMI had been stable over this time of period for our THA patients (Fig. 1, Table 3).

**Table 3:** Significance of Slopes

		**Slope (Pearson's r)**	**95% CI**	***P*-value**
**Overall BMI Trends**	**THA**	0.02	-0.07 to 0.11	0.617
	**Annual Physicals**	0.33	0.28 to 0.39	< 0.001
**Underweight**	**THA**	0.06	-0.03 to 0.16	0.156
	**Annual Physicals**	-0.16	-0.25 to -0.07	0.005
**Obese**	**THA**	-0.12	-1.08 to 0.84	0.772
	**Annual Physicals**	1.91	1.59 to 2.23	< 0.001
**Class I Obesity**	**THA**	-0.44	-1.37 to 0.50	0.296
	**Annual Physicals**	1.02	0.82 to 1.23	< 0.001
**Class II Obesity**	**THA**	0.15	-0.16 to 0.46	0.281
	**Annual Physicals**	0.55	0.46 to 0.64	< 0.001
**Class III Obesity**	**THA**	0.18	0.03 to 0.38	0.077
	**Annual Physicals**	0.33	0.28 to 0.38	< 0.001

After stratification into BMI categories, significant differences in the proportion of patients from each category were found each year between cohorts (*P* < 0.01). The proportion of underweight THA patients was significantly lower every year except 2018 and 2019, compared to the APG (Table 4). In contrast, the proportion of patients in the remaining four subdivisions was significantly higher for the THA group each year compared to the APG (Table 4).

**Table 4:** Proportion of Patients in Each Category

		# of patients (THA)	% of patients (THA)	# of patients (Annual Physicals)	% of patients (Annual Physicals)	*P*-value
**Underweight (BMI < 18.5)**	**2013**	5 (*n* = 832)	0.6	720 (*n* = 23,837)	3	< 0.001
	**2014**	10 (*n* = 1,109)	0.9	1,097 (*n* = 39,341)	2.8	< 0.001
	**2015**	18 (*n* = 1,379)	1.3	1,421 (*n* = 66,708)	2.1	0.035
	**2016**	19 (*n* = 1,584)	1.2	2,085 (*n* = 99,086)	2.1	0.01
	**2017**	18 (*n* = 1,709)	1.1	3,053 (*n* = 147,724)	2.1	0.003
	**2018**	26 (*n* = 1,777)	1.5	3,797 (*n* = 214,520)	1.8	0.328
	**2019**	20 (*n* = 1,547)	1.3	4,281 (*n* = 236,238)	1.8	0.127
	**2020**	13 (*n* = 1,313)	1	3,965 (*n* = 212,464)	1.9	0.019
**Obese (BMI ≥ 30)**	**2013**	357 (*n* = 832)	42.9	3,853 (*n* = 23,837)	16.2	< 0.001
	**2014**	441 (*n* = 1,109)	39.8	6,957 (*n* = 39,341)	17.7	< 0.001
	**2015**	529 (*n* = 1,379)	38.4	13,945 (*n* = 66,708)	20.9	< 0.001
	**2016**	595 (*n* = 1,584)	37.6	22,605 (*n* = 99,086)	22.8	< 0.001
	**2017**	620 (*n* = 1,709)	36.3	36,671 (*n* = 147,724)	24.8	< 0.001
	**2018**	657 (*n* = 1,777)	37	58,680 (*n* = 214,520)	27.4	< 0.001
	**2019**	639 (*n* = 1,547)	41.3	65,527 (*n* = 236,238)	27.7	< 0.001
	**2020**	541 (*n* = 1,313)	41.2	61,400 (*n* = 212,464)	28.9	< 0.001
**Class I Obesity (BMI: 30–34.9)**	**2013**	235 (*n* = 832)	28.2	2,505 (*n* = 23,837)	10.5	< 0.001
	**2014**	286 (*n* = 1,109)	25.8	4,524 (*n* = 39,341)	11.5	< 0.001
	**2015**	321 (*n* = 1,379)	23.3	8,867 (*n* = 66,708)	13.3	< 0.001
	**2016**	326 (*n* = 1,584)	20.6	14,264 (*n* = 99,086)	14.4	< 0.001
	**2017**	360 (*n* = 1,709)	21.1	22,823 (*n* = 147,724)	15.4	< 0.001
	**2018**	395 (*n* = 1,777)	22.2	35,886 (*n* = 214,520)	16.7	< 0.001
	**2019**	379 (*n* = 1,547)	24.5	39,801 (*n* = 236,238)	16.8	< 0.001
	**2020**	319 (*n* = 1,313)	24.3	37,024 (*n* = 212,464)	17.4	< 0.001
**Class II Obesity (BMI: 35–39.9)**	**2013**	85 (*n* = 832)	10.2	851 (*n* = 23,837)	3.6	< 0.001
	**2014**	97 (*n* = 1,109)	8.7	1,576 (*n* = 39,341)	4	< 0.001
	**2015**	143 (*n* = 1,379)	10.4	3,303 (*n* = 66,708)	5	< 0.001
	**2016**	173 (*n* = 1,584)	10.9	5,413 (*n* = 99,086)	5.5	< 0.001
	**2017**	182 (*n* = 1,709)	10.6	8,860 (*n* = 147,724)	6	< 0.001
	**2018**	164 (*n* = 1,777)	9.2	14,508 (*n* = 214,520)	6.8	< 0.001
	**2019**	168 (*n* = 1,547)	10.9	16,358 (*n* = 236,238)	6.9	< 0.001
	**2020**	145 (*n* = 1,313)	11	15,412 (*n* = 212,464)	7.3	< 0.001
**Class III Obesity (BMI ≥ 40)**	**2013**	37 (*n* = 832)	4.4	497 (*n* = 23,837)	2.1	< 0.001
	**2014**	58 (*n* = 1,109)	5.2	857 (*n* = 39,341)	2.2	< 0.001
	**2015**	65 (*n* = 1,379)	4.7	1,775 (*n* = 66,708)	2.7	< 0.001
	**2016**	96 (*n* = 1,584)	6.1	2,928 (*n* = 99,086)	3	< 0.001
	**2017**	78 (*n* = 1,709)	4.6	4,988 (*n* = 147,724)	3.4	0.007
	**2018**	98 (*n* = 1,777)	5.5	8,286 (*n* = 214,520)	3.9	< 0.001
	**2019**	92 (*n* = 1,547)	5.9	9,368 (*n* = 236,238)	4	< 0.001
	**2020**	77 (*n* = 1,313)	5.9	8,964 (*n* = 212,464)	4.2	0.003

**Fig. 2:**
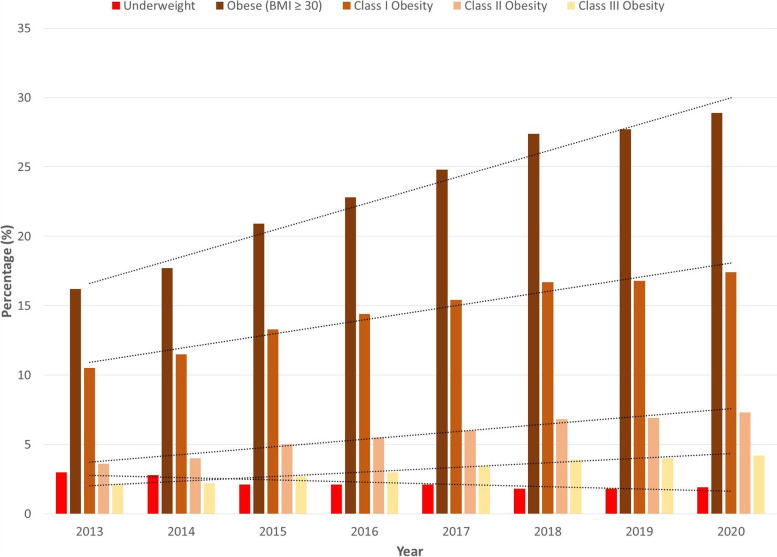
Proportion of Annual Physical Exam Patients

**Fig. 3:**
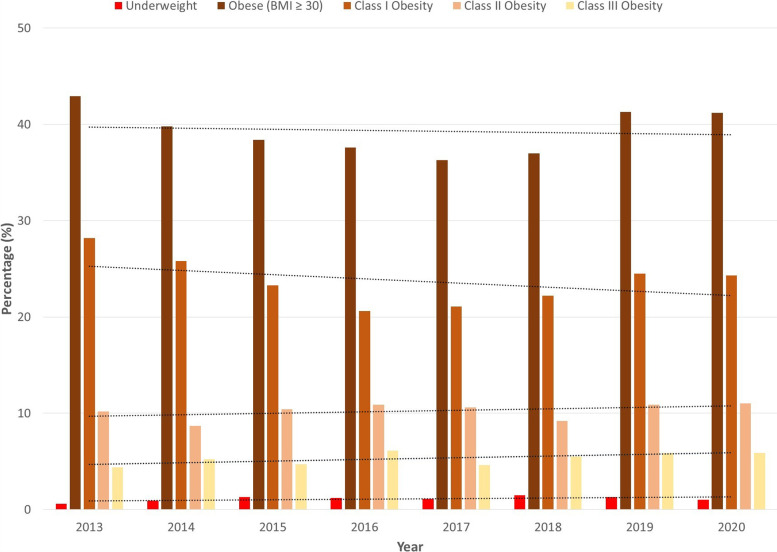
Proportion of THA Patients

Trend analysis of obesity rates for the five categories exhibited significance in all slopes for the APG (*P* < 0.001) and no significance for the THA group (Table 3). For the underweight category, there was a significant negative slope for the APG, but no significance was found for the THA group. This indicated progressively lower percentage of underweight patients in the APG over time, but no change for those undergoing THA (Figs. 2 and 3). In the obese, Class I, Class II, and Class III subdivisions, significant positive slopes, indicating progressively higher rate of obesity, were found for the APG, but not for THA patients (Figs. 2 and 3).

On analysis of matched age subgroups, we once again found significant differences in average BMI. Mean BMI for the THA group was significantly greater (*P* < 0.001) for each age range compared to the APG (Table 5)*,* which was similar to our non-matched results.

**Table 5:** BMI Comparison in Matched Age Subgroups (2013–2020)

Age (years.) Subgroup	THA	Annual Physicals	Unstandardized Beta (95% CI)	*P*-value
**20–29**	26.54 ± 6.79 (*n* = 95)	25.25 ± 5.64 (*n* = 191,054)	24.80 (23.65 to 25.95)	< 0.001
**30–39**	28.24 ± 5.93 (*n* = 275)	26.59 ± 5.95 (*n* = 219,986)	21.36 (20.60 to 22.11)	< 0.001
**40–49**	30.04 ± 6.51 (*n* = 899)	27.83 ± 6.07 (*n* = 200,808)	25.70 (25.12 to 26.27)	< 0.001
**50–59**	30.00 ± 6.27 (*n* = 2,643)	28.29 ± 5.98 (*n* = 220,612)	30.22 (29.69 to 30.75)	< 0.001
**60–69**	29.26 ± 6.04 (*n* = 4,001)	28.02 ± 5.72 (*n* = 141,086)	30.96 (30.21 to 31.72)	< 0.001
**70–79**	28.25 ± 5.52 (*n* = 2,591)	27.64 ± 5.35 (*n* = 32,493)	34.12 (32.62 to 35.63)	< 0.001
**80–89**	27.06 ± 4.75 (*n* = 692)	26.64 ± 4.87 (*n* = 8,890)	41.40 (38.40 to 44.40)	< 0.001

## Discussion

Obesity continues to be more prevalent in the United States, which contributes to the risk of developing osteoarthritis that leads to an increased demand for THA. Obesity rates are also steadily increasing, and, by 2030, are expected to exceed 50% [9]. Previous literature has shown an increase in obesity rates over time for patients undergoing THA, and that THA patients have a significantly higher average BMI compared to the overall United States population [5]. Additionally, patients with obesity may require THA at a younger age and are at a higher risk for perioperative complications after THA, including infection, wound complications, and aseptic loosening, which are more profound for THA *vs*. TKA [10–13]. Despite lower objective outcomes, obese patients are shown to benefit significantly from THA, as large studies demonstrated significant improvement after THA for all weight classes [14, 15]. Thus, more evidence is needed to evaluate protocols around THA in patients with obesity. The introduction of value-based payments in the most recent decade has shown an increased focus on such protocols like weight optimization and weight assessment for THA patients. Therefore, the goal of this study was to investigate change in obesity rates from 2013–2020 for patients receiving THA at a large urban academic health system relative to a population receiving annual physicals at the same institution. Our findings suggest that while THA patients are significantly more obese than the general population of patients, there have been stable trends in BMI and obesity rates over time among these patients compared to increasing trends seen in our general population.

Prior research has shown increasing prevalence of obesity in the United States, and that those who receive THA have higher BMI than the average American [5, 9]. Our findings corroborated this finding. Average BMI for the general patient population at our institution has steadily increased over the last decade. Those who received THA had higher average BMI than the APG group in every year studied. However, while prior studies have shown increase, over time, of average BMI for THA patients [16], our analysis showed that in the last eight years, there has been no significant positive or negative trend in obesity rates for our patients who undergo THA. In 2013, our institution was one of the first to enter into a value-based care contract through BPCI. These programs penalize institutions for poor outcomes and encourage preoperative optimization of modifiable risk factors like obesity. While there is substantial evidence for increased complications and worse outcomes for THA in patients with morbid obesity, studies have also shown that these patients benefit significantly from THA [17–19]. In this study period, our THA patients did not show the upward trend in prevalence of obesity demonstrated by historical data and our own general patient population over the same period. Although the average BMI among our THA was greater than the APG cohort at all-time points. Further study of the effects of optimization and future direction for THA in patients with obesity may be warranted.

A more granular analysis separating patients in to underweight, normal weight, Class I obesity, Class II obesity, and Class III obesity found proportion of THA patients in all five weight classes was stable during the study period. Conversely, the APG cohort had progressively higher proportions of patients in all obese classifications, and lower proportions of patients in the underweight category. Previous literature has shown that Class III obese THA patients have longer LOS and higher readmission and major complication rates than patients without obesity [20, 21]. A study by Fu *et al*. investigating THA in patients with obesity found that malnutrition is more prevalent in patients with Class III obesity than in Class I obesity, and portends worse outcomes than obesity itself [22]. Katakam *et al.* analyzed 1256 THAs and found that obesity Class III patients were 2.5 times more likely not to achieve minimal clinically relevant improvement in patient-reported outcome measures after surgery [23]. As a result, hospitals and surgeons will need to enhance optimization and patient selection for THA.

While previous literature has clearly linked obesity to worse outcomes and increased risk of complications after THA, there is less consensus regarding outcomes for underweight THA patients. Studies have shown that underweight patients who undergo primary THA require longer LOS and are readmitted more often than patients in normal weight categories, but they do not have higher rates of complications [24–26]. Possible explanations for these findings include prevalence of malnutrition among underweight patients [27]. Studies have also shown a relatively low rate of THA in patients who are underweight [15]. Our findings concurred with this result, as underweight patients were the only weight category studied where proportions were consistently lower for the THA cohort than for the APG cohort over the study period.

### Limitations

As a retrospective study extracted from electronic medical records, our data are limited, depending on accurate documentation such as ICD coding of physical exams and THA. Additionally, specifically selecting patients who have received an annual physical exam at our healthcare network to represent the general public may be biased towards those with more health access or may not account for those seeking care for a wide range of reasons, which could have influenced the lower obesity rates observed in our APG. The generalizability of our data is further limited due to the regional differences in obesity trends observed as the levels of obesity in our metropolitan urban area may differ from other areas of the country with higher or lower levels. Finally, the BMI of the general public may not be reflected in patients who self-select to undergo annual physical examinations. Despite these limitations, our observational study used sound design and statistical methodology, which, combined with access to a comprehensive patient record database, allows us to be confident in the reliability and validity of our data.

## Conclusion

Our study showed higher average BMI every year for THA patients *vs*. APG patients. However, contrary to prior studies, while BMI and obesity rates in each class for APG patients increased significantly over the eight-year period, there was no such trend among patients who received THA. This suggests that while our general patient population became increasingly obese, obesity rates among our THA patients since 2013 have remained flat. With access to this information, surgeons performing THA can consider if additional counseling for weight management is necessary, or if focus on optimization of other conditions is warranted prior to surgery. Furthermore, our data can inform institutional THA eligibility criteria for patients with obesity. Further research on the effect of value-based payment-driven optimization and patient selection efforts in THA is necessary.

## Data Availability

The datasets used and/or analyzed during the current study are available from the corresponding author on reasonable request.

## References

[1] George J, Klika AK, Navale SM (2017). Obesity Epidemic: Is Its Impact on Total Joint Arthroplasty Underestimated? An Analysis of National Trends. Clin Orthop Relat Res.

[2] Hales CM, Carroll MD, Fryar CD, Ogden CL. Prevalence of obesity and severe obesity among adults: United States, 2017-2018. NCHS Data Brief. 2020;360:1–8. 32487284

[3] Bourne R, Mukhi S, Zhu N (2007). Role of obesity on the risk for total hip or knee arthroplasty. Clin Orthop Relat Res.

[4] Onggo JR, Onggo JD, de Steiger R (2020). Greater risks of complications, infections, and revisions in the obese versus non-obese total hip arthroplasty population of 2,190,824 patients: a meta-analysis and systematic review. Osteoarthr Cartil.

[5] Pirruccio K, Sloan M, Sheth NP. Trends in obesity prevalence among total hip arthroplasty patients and the effect on surgical outcomes, 2008–2016. J Orthop;16. Epub ahead of print 2019. 10.1016/j.jor.2019.03.024. 10.1016/j.jor.2019.03.024 PMC6458338 31007457

[6] Johnson C, White C, Kunkle B (2021). Effects of the Obesity Epidemic on Total Hip and Knee Arthroplasty Demographics. J Arthroplasty.

[7] Singh J, Lewallen D. Increasing obesity and comorbidity in patients undergoing primary total hip arthroplasty in the U.S.: a 13-year study of time trends. BMC Musculoskelet Disord;15. Epub ahead of print 2014. 10.1186/1471-2474-15-441. 10.1186/1471-2474-15-441 PMC4302153 25519434

[8] Centers for Disease Control and Prevention. Defining adult overweight & obesity. U.S Department of Health and Human Services; 2022. https://www.cdc.gov/obesity/basics/adult-defining.html.

[9] Wang Y, Beydoun MA, Min J (2020). Has the prevalence of overweight, obesity and central obesity levelled off in the United States? Trends, patterns, disparities, and future projections for the obesity epidemic. Int J Epidemiol.

[10] Haynes J, Nam D, Barrack RL (2017). Obesity in total hip arthroplasty: does it make a difference?. Bone Joint J.

[11] Goodnough L, Finlay A, Huddleston J (2018). Obesity Is Independently Associated With Early Aseptic Loosening in Primary Total Hip Arthroplasty. J Arthroplasty.

[12] Kong L, Cao J, Zhang Y (2017). Risk factors for periprosthetic joint infection following primary total hip or knee arthroplasty: a meta-analysis. Int Wound J.

[13] DeMik DE, Bedard NA, Dowdle SB (2018). Complications and Obesity in Arthroplasty—A Hip is Not a Knee. J Arthroplasty.

[14] Haebich S, Mark P, Khan R (2020). The Influence of Obesity on Hip Pain, Function, and Satisfaction 10 Years Following Total Hip Arthroplasty. J Arthroplasty.

[15] Mukka S, Rolfson O, Mohaddes M (2020). The Effect of Body Mass Index Class on Patient-Reported Health-Related Quality of Life Before and After Total Hip Arthroplasty for Osteoarthritis: Registry-Based Cohort Study of 64,055 Patients. JBJS Open Access.

[16] Etcheson J, George N, Gwam C (2018). Trends in Total Hip Arthroplasty Under the Patient Protection and Affordable Care Act: A National Database Analysis Between 2008 and 2015. Orthopedics.

[17] Kim KY, Anoushiravani AA, Chen KK (2019). Perioperative Orthopedic Surgical Home: Optimizing Total Joint Arthroplasty Candidates and Preventing Readmission. J Arthroplasty.

[18] Feng JE, Novikov D, Anoushiravani AA (2018). Team Approach: Perioperative Optimization for Total Joint Arthroplasty. JBJS Rev.

[19] Boraiah S, Joo L, Inneh IA (2015). Management of Modifiable Risk Factors Prior to Primary Hip and Knee Arthroplasty. J Bone Jt Surgery-American.

[20] Rajgopal R, Martin R, Howard J (2013). Outcomes and complications of total hip replacement in super-obese patients. Bone Joint J.

[21] Schwarzkopf R, Thompson SL, Adwar SJ (2012). Postoperative Complication Rates in the ‘Super-Obese’ Hip and Knee Arthroplasty Population. J Arthroplasty.

[22] Fu MC, McLawhorn AS, Padgett DE (2017). Hypoalbuminemia Is a Better Predictor than Obesity of Complications After Total Knee Arthroplasty: a Propensity Score-Adjusted Observational Analysis. HSS J.

[23] Katakam A, Florissi IS, Colon Iban YE (2021). Class III Obesity Increases Risk of Failure to Achieve the 1-Year Hip Disability and Osteoarthritis Outcome Score-Physical Function Short Form Minimal Clinically Important Difference Following Total Hip Arthroplasty. J Arthroplasty.

[24] Zusmanovich M, Kester B, Feng J (2018). Postoperative complications in underweight patients undergoing total hip arthroplasty: a comparative analysis to normal weight patients. J Orthop.

[25] Saucedo JM, Marecek GS, Wanke TR, et al. Understanding readmission after primary total hip and knee arthroplasty: Who’s at risk? J Arthroplasty;29. Epub ahead of print 2014. 10.1016/j.arth.2013.06.003. 10.1016/j.arth.2013.06.003 23958236

[26] Katakam A, Melnic C, Bragdon C (2021). Low Body Mass Index Is a Predictor for Mortality and Increased Length of Stay Following Total Joint Arthroplasty. J Arthroplasty.

[27] Ihle C, Freude T, Bahrs C (2017). Malnutrition - An underestimated factor in the inpatient treatment of traumatology and orthopedic patients: a prospective evaluation of 1055 patients. Injury.

